# Excess mortality after hip fracture during COVID-19 pandemic: More about disruption, less about virulence—Lesson from a trauma center

**DOI:** 10.1371/journal.pone.0263680

**Published:** 2022-02-25

**Authors:** Baptiste Boukebous, Cédric Maillot, Angèle Neouze, Hélène Esnault, Fei Gao, David Biau, Marc-Antoine Rousseau

**Affiliations:** 1 Department of Orthopaedic and Traumatological Surgery, Beaujon/Bichat Hospitals, APHP.Nord University of Paris, Clichy, France; 2 ECAMO Team, INSERM, UMR1153, Centre of Research in Epidemiology and StatisticS, Hôtel-Dieu Hospital, Paris, France; 3 Department of Geriatrics, Bichat Hospital, APHP.Nord University of Paris, Paris, France; 4 REPERES Team, École des hautes études en santé publique, Rennes, France; 5 Department of Orthopaedic and Traumatological Surgery, Cochin Hospital, APHP, University of Paris, Paris, France; Medical College of Wisconsin, UNITED STATES

## Abstract

To date, literature has depicted an increase in mortality among patients with hip fractures, directly related to acute coronavirus disease 2019 (COVID-19) infection and not due to underlying comorbidities. Usual orthogeriatric pathway in our Department was disrupted during the pandemic. This study aimed to evaluate early mortality within 30 days, in 2019 and 2020 in our Level 1 trauma-center. We compared two groups of patients aged >60 years, with osteoporotic upper hip fractures, in February/March/April 2020 and February/March/April 2019, in our level 1 trauma center. A total of 102 and 79 patients met the eligibility criteria in 2019 and 2020, respectively. Mortality was evaluated, merging our database with the French open database for death from the INSEE, which is prospectively updated each month. Causes of death were recorded. Charlson Comorbidity Index was evaluated for comorbidities, Instrumental Activity of Daily Living (IADL), and Activity of Daily Living (ADL) scores were assessed for autonomy. There were no differences in age, sex, fracture type, Charlson Comorbidity Index, IADL, and ADL. 19 patients developed COVID-19 infection. The 30-day survival was 97% (95% CI, 94%–100%) in 2019 and 86% (95% CI, 79%–94%) in 2020 (HR = 5, 95%CI, 1.4–18.2, p = 0.013). In multivariable Cox’PH model, the period (2019/2020) was significantly associated to the 30-day mortality (HR = 6.4, 95%CI, 1.7–23, p = 0.005) and 6-month mortality (HR = 3.4, 95%CI, 1.2–9.2, p = 0.01). COVID infection did not modify significantly the 30-day and 6-month mortality. This series brought new important information, early mortality significantly increased because of underlying disease decompensation. Minimal comprehensive care should be maintained in all circumstances in order to avoid excess of mortality among elderly population with hip fractures.

## Introduction

Since December 2019, the new coronavirus disease 2019 (COVID-19) has spread worldwide and led to successive confinements of populations. The COVID-19 disease stems from animal transmission but overall, several other emerging viral zoonoses already exist [1, 2], and that can possibly bring about similar global pandemics. A careful assessment of mortality is mandatory because lessons from the COVID-19 pandemic may be useful in case of a future new pandemic.

Regarding trauma, the epidemiology of trauma changed during confinements with the decrease in the incidence of road or sports accidents, but an increase in the number of household accidents and domestic violence-related injuries [3]. However, hip fractures are specific because they affect in-home fall [4] mechanisms in the elderly population with low energy. Hip fracture incidence was stable over time in level 1 trauma centers in China [4], Spain [5], and the United Kingdom [6, 7], and the specific pathway flow was established to continue the treatment of these fractures. The principle of these pathways is to separate patients with COVID-19 from those without to limit transmission and treat COVID-19 symptoms.

Elderly fragility and institutionalization increase the risk of COVID-19 in patients with hip fractures [8]. Having said that, it becomes interesting to assess whether the excess of mortality in institutions during the pandemic is due to COVID-19 infection or baseline frailty. In New York, Egol et al. found similar baseline comorbidities between patients with and without COVID-19 and the 30-day mortality was equivalent in patients without infection in 2020, in comparison with patients in the same centers in 2019 [9]. All in all, the increase in mortality among patients with hip fractures has seemed to be directly related to acute COVID-19 infection [4–7, 9–11] and not due to underlying comorbidities so far.

In France, the first patients with COVID-19 appeared at the end of January 2020, but few other cases were suspected without biological confirmation by the end of 2019. The first death due to COVID-19 in France occurred in mid-February, and the first clusters were identified in a military base in the north of Paris by the end of February. At this time, our hospital was the first reference center for COVID-19 in Paris with several department reorganizations. A first confinement started on March 17, 2020 and was extended until May 11, 2020. A total of 26,643 persons died of COVID19 during this first period.

In contrast to the data found in the literature, the incidence of hip fractures in our level 1 trauma center decreased during confinement. Furthermore, although our department is responsible for musculoskeletal care in an area of at least 410,000 inhabitants, the incidence of patients with COVID-19 infection and hip fracture was lower than of those from other countries. Our Department provides orthogeriatric care and this pathway was seriously disrupted during confinement because the two geriatricians who used to be fully committed in the orthopedic ward were transferred to another department. This is a relevant data because literature shows that orthogeriatric pathways are responsible for decreasing postoperative mortality [12–14]. Moreover, the benefits of orthogeriatric comprehensive care seem to be the most important for autonomous and healthy elderly patients [13]. In such a situation, regardless of their COVID-19 status, the disruption of our orthogeriatric chain likely affected the outcomes for all patients with hip fracture. Such an increase in mortality about disruption had already been described in other specialties.

For all these reasons, mortality outcomes should not be only evaluated in a confinement period cohort, comparing patients with or without COVID-19, because actual mortality should be minimized. A comparison with an anterior cohort from a similar period with exhaustive medical and social evaluation appears to be mandatory. There are still poor comparative data for mortality in literature with such a methodology and orthogeriatric center.

This study aimed to evaluate the 30-day mortality in 2019 and 2020, whereas the COVID-19 epidemic was fully active in France with confinement. The main hypothesis was that the recruitment periods contributed significantly to early mortality risk.

## Methods

This work has been reported in line with the STROCSS criteria.

### Study design

This was a historical cohort study that was conducted to compare the 30-day mortality in the two groups of patients aged >60 years with osteoporotic upper hip fracture. The first group named *2020* included patients with fractures during the COVID-19 epidemic peak in France, and the second group called *2019* involved patients with the same fractures, treated in our department in 2019. According to the AO classification, fractures are defined as trochanteric region fractures (AO 31A) or femur neck fractures (AO 31B).

The period of recruitment began on February 1 and ended April 30 in both 2019 and 2020 groups. Follow-up was at least 30-day for all patients, and the last update for death assessment was October 1, 2020. The index date for the calculation of the postoperative follow-up was the date of the surgery.

### Setting

All patients were treated in our orthopedic department, which is a level 1 trauma center within a university hospital complex. The department is divided into two sites, separated approximately 2 km from each other. Two geriatricians are usually fully committed in the orthopedic ward and provide comprehensive care for the patients aged over 70 years, preoperatively and postoperatively. Still, they were transferred back to the Geriatric department during the breakthrough. The 14 surgeons of our team all work in both places. Under normal conditions, the very large majority of hip fractures are treated in our orthogeriatric unit with the help of the two geriatricians. During the confinement, all elective surgeries were cancelled, and every operative theater were dedicated for emergencies, resulting in a decrease in the delays for traumas. The indications were not modified, and there was still a service meeting every morning. The patients with hip fractures also infected with COVID-19 were admitted to a dedicated medical ward and the surgeries were performed in a dedicated operating room as soon as possible. Patients came from 22 cities around the hospital, among which six cities, totaling 410,000 inhabitants according to the French National Institute for Demography [15] (INSEE, *Institut National de la Statistique et des Etudes Economiques*), do not have any other trauma center.

### Participants

The eligibility criterion was AO31A or AO31B fracture of the upper femur with surgical indication. Patients were sought in the operating room legers. In eligible patients, medical records were opened using Orbis software (Agfa HealthCare Corporation, Greenville, South Carolina, USA), which is the common medical software in all hospitals of our institution. Patients with pathologic fractures and high-energy polytrauma were excluded and such information were found in the medical records.

102 upper femur fractures in 2019 and 79 fractures in 2020, met the eligibility criteria.

### Primary outcome

The primary outcome was the 30-day survival.

We merged the database of included patients with the French open database for death from INSEE [16], which lists all deaths of French citizens from 1970 and is prospectively updated each month. This is a free open-access database, available on *https*:*//deces*.*matchid*.*io/search*. Any other person can access this database, on the same basis that the authors, but investigation requires the full identity of patients (family name, given names, date of birth, hometown). Merging was performed, concatenating surname, name, and date of birth. This method allowed the assessment of all deaths, and if death occurred, the date is determined. Survival was the time spent from the day of the surgery. The INSEE database is exhaustive, so there were no censored patients up to the follow-up time, regarding death, due to missing data. The minimal anonymized dataset related to this study is available online on *https*:*//github*.*com/bboukebous/fractureCovid*.

Patients could either be alive or dead up to 30-day. The cause of death was recorded. If it occurred in our hospitals, medical record review on Orbis software allowed the assessment of the cause of death. If not, the family physician is called back.

### Variables and sources

Medical and social variables were collected, extracting data from medical records on Orbis software, including age, sex, medical history, place of residence, delay of surgery, and delay of orthopedic department discharge. The weight of comorbidities was evaluated by calculating the Charlson Comorbidity Index. All the comorbidities were systematically carefully recorded preoperatively and notified in the medical record by the anesthesiologist. We used the website MDcalc [17] to operate the scores. The autonomy of patients was evaluated by calculation of the mean of Instrumental Activities of Daily Living (IADL) and Activity of Daily Living (ADL) scores and the determination of the type of residence, which could be proper or institutional residence. Infection with COVID was also reported, all patients admitted to our ward received a PCR test in 2020. In case of any doubt about an ancient infection, a serological test was also carried out. Postoperative COVID infections up to one year were reported using the medical records or phoning the patients in case of doubt. Medical and surgical complications were evaluated. Distance, in a straight line, between the place of residence and hospital was calculated by transforming address into GPS coordinates using the French government database for cities [18] and the formula provided in the annex.

The 6-month mortality was a secondary outcome.

### Statistical methods and bias

When describing the database, for quantitative variables, we reported means and standard deviations. We reported counts for categorical variables. Differences in medical and social outcomes between 2019 and 2020 were analyzed using the Student test for quantitative variables and the Chi-square test for qualitative variables.

There are no missing values for the death variable. We counted missing values for medical and social variables. We used a multiple imputation using Multivariate Imputation by Chained Equation (MICE) approach for variables with up to 30% of missing data.

The main hypothesis was that the recruitment periods (2019 or 2020) contributed significantly to 30-day mortality risk (the primary outcome).

We compared the 30-day mortality between the two periods using an unadjusted Cox’s proportional hazards (Cox’s PH) model. Then, a multivariable Cox’s PH regression was carried to account for potential confounders. Confounders were selected based on their association with the primary outcome in unadjusted Cox’s PH regression models with a cut-off of 0.2.

The period was automatically incorporated within the explicative variables of the multivariable regression. The Charlson score was also automatically included in the final multivariable-adjusted model because it is well-known risk factors for mortality. The hazard ratios (HR) with their confident interval were assessed for the variables which appeared significant in the model. We repeated the process in a second regression to assess the 6-month mortality. To consider the family-wise error with this second regression, we corrected the alpha risk using the Bonferroni method, and we accepted significance with a p-value less than 0.025 (0.05/2). The proportional hazard assumptions were accepted if non-significant associations (p>0.05) were found between the residuals of the regressions and the time; the function *cox*.*zph()* of the R software’s *survival* package was used.

Analyses were performed using the R software (R 3.3.0, R Foundation for Statistical Computing).

This study ensured the confidentiality of personal data in accordance with the MR-004 form of the *French National Commission for Data Protection Commission* and has registration number **2219604**. It was also registered under the number NCT04916561 in ClinicalTrials.gov.

## Results

### Descriptive data

Table 1 details the demographic and medical characteristics of the participants. Two groups did not present any difference in age, sex, fracture type, Charlson Comorbidity Index, ADL, IADL, and dementia rate. Eight of 79 patients in 2020 and three of 102 patients in 2019 were nonoperatively treated (p = 0.09). The average Charlson Comorbidity Index and ADL score for nonoperatively treated patients were 7.7 and 4, respectively, versus 6 (p = 0.02) and 4.7 (p = 0.2), respectively, for others.

**Table 1:** Demographic and medical characteristic of the cohort.

Variable	2019	2020	Overall	p.value
	(N = 102)	(N = 79)	(N = 181)	
**Age**				0.778
Count	102	79	181	
Mean (SD)	84.04 (9.24)	83.63 (9.89)	83.86 (9.51)	
**Sex**				0.353
Count (Row %)	102	79	181	
female (Col %)	76 (75%)	53 (67%)	129 (71%)	
**Month**				0.038
Count (Row %)	102	79	181	
February (Col %)	35 (34%)	31 (39%)	66 (36%)	
March (Col %)	29 (28%)	32 (41%)	61 (34%)	
April (Col %)	38 (37%)	16 (20%)	54 (30%)	
**Nonoperatively treates**				0.09
Count (Row %)	102	79	181	
yes (Col %)	3 (3%)	8 (10%)	11 (6%)	
**Charlson**				0.789
Count	98	73	171	
Mean (SD)	6.18 (2.26)	6.08 (2.59)	6.14 (2.40)	
Missing	4	6	10	
**IADL**				0.694
Count	75	44	119	
Mean (SD)	1.83 (1.57)	1.98 (2.10)	1.89 (1.77)	
Missing	27	35	62	
**ADL**				0.217
Count	83	53	136	
Mean (SD)	4.58 (1.78)	4.96 (1.70)	4.73 (1.75)	
Missing	19	24	43	
**Habitation**				0.506
Count (Row %)	102	78	180	
institution (Col %)	16 (16%)	13 (17%)	29 (16%)	
home (Col %)	86 (84%)	64 (82%)	150 (83%)	
Missing	0	1	1	
**Dementia**				0.468
Count (Row %)	102	78	180	
yes (Col %)	44 (43%)	31 (40%)	75 (42%)	
Missing	0	1	1	
**COVID infection**				<0.001
Count (Row %)	102	79	181	
yes (Col %)	0 (0%)	19 (24%)	19 (10%)	
**Death**				0.416
Count (Row %)	102	79	181	
yes (Col %)	16 (16%)	17 (22%)	33 (18%)	
**Death by 30 days**				0.014
Count (Row %)	102	79	181	
yes (Col %)	3 (3%)	11 (14%)	14 (8%)	

The difference between the two time periods was assessed using Student t’s test for continuous variables or chi-squared test for categorical variables.

Comparative Kaplan Meier curves for the overall 30-day mortality, in 2019 and 2020.

In 2019, five patients came from other regions or countries. When considering the 93 patients in 2019 from our region, the average distance between hospital and place or habitation was 3.66 km, the average distance in 2020 was 3.5 km, and remoteness was not significantly different between the two periods.

The average delay of surgery was 3 days in 2019 and 1.8 days in 2020 (p = 0.0002).

### Main results

**Fig 1:**
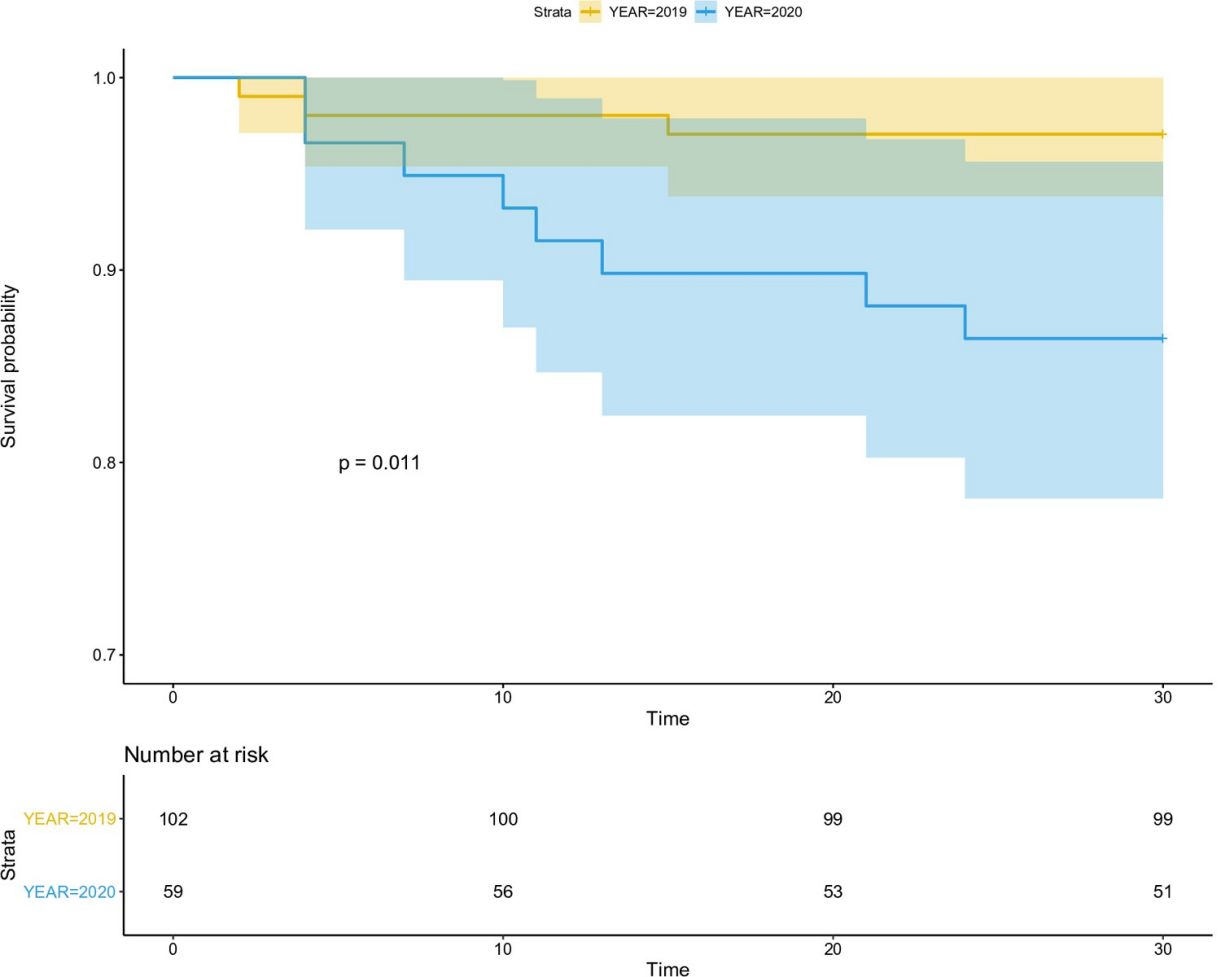
Comparative Kaplan Meier curves for the overall 30-day mortality, in 2019 and 2020. Significant difference in the 30-day mortality between 2019 and 2020.

A total of 42 patients died, the overall 30-day survival was 92% (95% CI, 88%–96%), and the overall 6-month survival was 78% (95% CI, 78%–89%). In 2019, three patients died before 30-day postoperatively, and survival was 97% (95% CI, 94%–100%). In 2020, 11 patients died before 30-day postoperatively, and the survival rate was 86% (95% CI, 79%–94%). The difference between the two periods was significant (HR = 5, 95%CI, 1.4–18.2, p = 0.013) (Fig 1). The 6-month survival was also lesser in 2020 compared to 2019 (HR = 2.3, 95%CI, 1.1–5, p = 0.023). The 1-year survival for patients of the 2019 group was 84% (95% CI, 78%–89%).

19 patients had COVID-19 in 2020, either in the same period as acquiring fracture or afterward. In 2020, COVID-19 was the cause of the deaths of four of 19 patients, and three patients within 30-day. All causes for the two periods are presented in Table 2. Number of deaths due to bed ridden tended to be higher in 2019 than 2020 (6.8% versus 1.3%, p = 0.14). Among the seven patients who were nonoperatively treated in 2020, five did not die before October 1, 2020, and one of three patients nonoperatively treated in 2019 was still alive in 2020.

**Table 2:** Medical decompensations and cause of death.

Variable	2019	2020
**Medical complications**		
Count (Row %)	102 (57%)	77 (43%)
bacterial pneumonia (Col %)	3 (3%)	0 (0%)
brain acute hematoma (Col %)	2 (2%)	0 (0%)
shock (Col %)	0 (0%)	1 (1%)
COVID (postoperatively) (Col %)	-	7 (9%)
death, unknown cause (Col %)	1 (1%)	0 (0%)
decompensation: heart (Col %)	5 (5%)	2 (3%)
decompensation: kidney (Col %)	4 (4%)	1 (1%)
decompensation: liver (Col %)	0 (0%)	1 (1%)
decompensation: lungs (Col %)	0 (0%)	2 (3%)
heart attack (Col %)	0 (0%)	1 (1%)
influenza pneumonia (Col %)	2 (2%)	0 (0%)
pancreatic cancer (Col %)	0 (0%)	1 (1%)
pancreatitis (Col %)	1 (1%)	0 (0%)
peritonitis (Col %)	0 (0%)	1 (1%)
pulmonary embolism (Col %)	1 (1%)	0 (0%)
viral infection (non COVID) (Col%)	1 (1%)	0 (0%)
**Cause of deaths**		
Count (Row %)	102 (57%)	77 (43%)
acute brain hematoma (Col %)	1 (1%)	1 (1%)
bed ridden (Col %)	7 (7%)	1 (1%)
cancer (Col %)	2 (2%)	2 (3%)
shock (Col %)	-	2 (3%)
COVID (Col %)	-	4 (5%)
Bacterial pneumonia (Col %)	1 (1%)	-
decompensation: heart (Col %)	1 (1%)	4 (5%)
decompensation: kidney (Col %)	1 (1%)	1 (1%)
decompensation: liver (Col %)	-	1 (1%)
heart attack (Col %)	-	1 (1%)
perforated ulcere (Col %)	3 (3%)	1 (1%)

The Charlson comorbidity Index score, age, dementia, diagnosis, and delay of surgery did not significantly influence the mortality. The following factors were associated with the 30-day mortality in the unadjusted analyses: the sex (HR = 3.44, 95%CI, 1.2–10, p = 0.01) and the ADL score (HR = 0.69, 95%CI, 0.5–0.9, p = 0.006). The IADL variable was included in the multivariable model once imputed (Table 3). The COVID infection was also selected for the 30-day and 6-month multivariable models (HR = 2.5, 95%CI, 0.7–9, p = 0.15 at 30-day and HR = 2.2, 95%CI, 0.8–5.7, p = 0.1 at 6-month). In the multivariable Cox’s PH regression, the period was still significantly associated with the 30-day mortality (HR = 8.7, 95%CI, 2.15–35, p = 0.002) and COVID infection was not significant (Table 3).

**Table 3:** Unadjusted and multivariable-adjusted Cox’s proportional hazard regressions for the primary outcome 30-day mortality.

Parameter	Unadjusted			Multivariable-adjusted		
	HR	95% CI	p-value	HR	95% CI	p-value
**Period**	5	1.4–18	0.013	8.7	2.15–35	0.002
***Period*** *	*-*	*-*	*-*	*5*.*5*	*1*.*44–21*	*0*.*01*
**Charlson**	1.11	0.93–1.33	0.217	1.04	0.82–1.32	0.69
***Charlson*** *	*1*.*09*	*0*.*92–1*.*28*	*0*.*312*	*0*.*96*	*0*.*79–1*.*16*	*0*.*68*
**Sex (h)**	3.44	1.19–9.9	0.01	1.13	0.98–10	0.052
***Sex (h)*** *	*-*	*-*	*-*	*3*.*5*	*1*.*2–10*	*0*.*022*
**Age**	1.03	0.96–1.09	1	-	-	-
**ADL**	0.69	0.53–0.9	0.006	0.6	0.45–0.82	0.001
***ADL*** *	*0*.*68*	*0*.*52–0*.*88*	*0*.*004*	*0*.*5*	*0*.*37–0*.*87*	*0*.*009*
**IADL**	0.78	0.53–1.15	0.4	-	-	-
***IADL*** *	*0*.*79*	*0*.*55–1*.*12*	*0*.*18*	*1*.*9*	*0*.*69–1*.*91*	*0*.*57*
**COVID infection**	2.5	0.7–9	0.15	0.44	0.1–1.9	0.28
***COVID infection*** *	*-*	*-*	*-*	*0*.*68*	*0*.*16–2*.*90*	*0*.*63*
**Delay**	0.89	0.65–1.24	0.52	-	-	-
**Diagnosis**£	1.4	0.47–4.1	0.54	-	-	-

Parameters with a p-value less than 0.2 in the unadjusted analysis were included in the multivariable Cox’s PH regression. £ diagnosis either AO31A or AO31B fracture.

* results after Multivariate Imputation by Chained Equation (MICE) approach.

The 6-month mortality was significantly different between 2019 and 2020 (HR = 3.4, 95%CI, 1.2–9.2, p = 0.01) and the COVID infection did not interact significantly (p = 0.5). The proportional hazard assumptions were all accepted (p>0.05).

## Discussion

Fractures significantly decreased from 102 in 2019 to 79 in 2020 in our center. The present study mainly aimed to evaluate the extent to which the period modified mortality after upper femur fracture, in our orthopedic department. The main results highlight that the 2020 period was associated with an increase in the 30-day and 6-month mortality, although patients did not significantly have more comorbidities. Mortality over risk in 2020 remained true in the multivariable models, and COVID-19 infection did not significantly affect mortality. Also observed in 2020 was a non-significant increased trend for nonoperative treatment. The delay of surgery was significantly lesser in 2020.

To the best of our knowledge, independent of COVID-19 infection, this comparative series is the first to highlight an increase in mortality during the COVID-19 pandemic. The causes of death were mostly related to the systemic decompensation of underlying diseases. The total disruption of our comprehensive pathway with orthogeriatric care is our main hypothesis to explain this surge in mortality, independent of COVID-19. At the beginning of the pandemic, several publications proposed new organizations to manage COVID-19 patients and separate them from other emergencies [19–24]. After a few times, several surgical and medical specialties, such as bacteriologists, neurologists and oncologists, warned about the side effects of usual medical pathway disorganization [25–28].

Under normal circumstances, geriatricians are present in the care ward, and the very part of hip fractures are transferred here. Geriatrician assist surgeons with managing comorbidities and providing early treatment in case of perioperative complications. Geriatricians and surgeons also work together by examining all the patients who are hospitalized in the ward on a daily basis. Patients would be discharged from the department and placed in rehabilitation centers according to the complexity of the care they needed.

However, during the COVID peak period, geriatricians and surgeons were obliged to work in separate wards in order to participate to COVID infection care. The orthogeriatric ward closed and all trauma were treated at the hospital which normally receives polytraumatized patients, under normal conditions. As a result, daily visits with geriatricians were no longer possible and medical support was not offered automatically. The elective surgeries were canceled, every operating rooms were dedicated for emergencies so the delay between the admission and the operation decreased in 2020. In addition, the fact that many rehabilitation centers had been closed meant that patients were often discharged to non-specific geriatric reeducation centers, which did not necessarily have the resources needed to provide the level of care available previously. Nevertheless, the vast majority of the 30-day deaths in 2020 occurred in our orthopedic department, and the rehabilitation centers did not seem to be involved in the surge of mortality.

We identified four series on hip fractures comparing the 2020 period to previous periods, in which the crude 30-day mortality rates ranged from 2.2% to 8.6% but superior to 4% in most cases [9, 29–31]. In our series, the crude 30-day mortality rate in 2019 was 3 of 102 (2.9%), corresponding to a 3% mortality rate with Kaplan–Meier methods, which represents the lowest rate compared with that in other series. Furthermore, our crude 30-day rate is extremely close to that observed by Lau et al. with an orthogeriatric clinical pathway [12]. The 1 year crude mortality was also lower in 2019 than that in other orthogeriatric surveys [32]. For all these reasons, a significant early mortality observed in the 2020 period may correspond to cumulative effects of the loss of orthogeriatric care, which is responsible for underlying disease decompensations and COVID-19 infection, although this last reason did not show a significant level.

The main limitation of this series is the low number of patients with COVID-19 in 2020, leading to a lack of power to demonstrate the significant impact of COVID-19 on mortality. Based on Kayani et al.’s results [33], the 30-day mortality would increase from 10% to 30% in the case of COVID-19 infection in patients with hip fracture, indicating that our retrospective power would be approximately 50%. The Bonferroni method used to take into account the family-wise error exposed to an increase in the type two error risk but, in our case, the two p-value were less than 0.025. Thus, we maximized the chances to reduce the type one error without rejecting any hypothesis. Another limitation involves medical record filling that has poorer quality in 2020 than that in 2019, especially because of comprehensive care disruption. This loss in quality was responsible for more missing values regarding the Charlson Comorbidity Index, ADL, and IADL in 2020, compared with those in 2019. The Charlson Comorbidity Index can be easily reconstructed, whereas ADL and IADL cannot, and missing values were the main source of bias. Nevertheless, the results after the MICE approach were consistent with the primary ones, indicating that the findings were robust. Another limitation is the difference in the number of fractures between the two periods, which decreased from 102 to 79 without certified explanations. Several hypotheses stand out to explain such a difference: decrease in trauma incidence, migration of the population outside the urban area during confinement, unusual transfer of emergencies into private facilities, some patients with hip fracture could have die in their institution and have not been transferred to orthopedic departments.

## Conclusion

This series supports all older cases highlighting the increase in mortality after hip fracture during the COVID-19 pandemic peak. However, new information is essential in the 30-day mortality due to underlying disease decompensation, whereas COVID-19 infection did not reach a significant and independent level. The main hypothesis is that orthogeriatric pathways were totally disrupted compared with those in 2019, and such comprehensive care was used to provide small 30-day and 1-year mortality baseline rates. We warn doctors and health authorities that they should do their best in order to maintain the usual clinical pathways in all circumstances in order to avoid excess of mortality among elderly trauma patients in case of a new pandemic breakthrough. A retrospective assessment of the human resources and organization should be launched to provide a road map of actions for the future.
